# A unique life-strategy of an endophytic yeast *Rhodotorula mucilaginosa* JGTA-S1*—*a comparative genomics viewpoint

**DOI:** 10.1093/dnares/dsy044

**Published:** 2019-01-07

**Authors:** Diya Sen, Karnelia Paul, Chinmay Saha, Gairik Mukherjee, Mayurakshi Nag, Samrat Ghosh, Abhishek Das, Anindita Seal, Sucheta Tripathy

**Affiliations:** 1 Structural Biology and Bioinformatics Division, CSIR Indian Institute of Chemical Biology, Kolkata, India; 2 Department of Biotechnology, Dr. B. C. Guha Centre for Genetic Engineering and Biotechnology, University of Calcutta, Kolkata, India; 3 Department of Endocrinology & Metabolism, Institute of Post Graduate Medical Education & Research and SSKM Hospital, Kolkata, West Bengal, India; 4 Academy of Scientific and Innovative Research, New Delhi, India

**Keywords:** genome assembly, oxford nanopore, endophyte, *Rhodotorula*, endosymbiont

## Abstract

Endophytic yeasts of genus *Rhodotorula* are gaining importance for their ability to improve plant growth. The nature of their interaction with plants, however, remains unknown. *Rhodotorula mucilaginosa* JGTA-S1 was isolated as an endophyte of *Typha angustifolia* and promoted growth in the host. To investigate the life-strategy of the yeast from a genomics perspective, we used Illumina and Oxford Nanopore reads to generate a high-quality annotated draft assembly of JGTA-S1 and compared its genome to three other *Rhodotorula* yeasts and the close relative *Rhodosporidium toruloides*. JGTA-S1 is a haploid yeast possessing several genes potentially facilitating its endophytic lifestyle such as those responsible for solubilizing phosphate and producing phytohormones. An intact mating-locus in JGTA-S1 raised the possibility of a yet unknown sexual reproductive cycle in *Rhodotorula* yeasts. Additionally, JGTA-S1 had functional anti-freezing genes and was also unique in lacking a functional nitrate-assimilation pathway—a feature that is associated with obligate biotrophs. Nitrogen-fixing endobacteria were found within JGTA-S1 that may circumvent this defective N-metabolism. JGTA-S1 genome data coupled with experimental evidence give us an insight into the nature of its beneficial interaction with plants.

## 1. Introduction

*Rhodotorula* is a group of yeasts belonging to the phylum Basidiomycota, family Sporidiobolaceae, and class Microbotryomycetes. These are found from widely varying environmental sources ranging from extreme climates of deep-sea vents to arctic cold deserts^1^^,^^2^ from the soil, air, or as organisms associated with plants. Clinically *Rhodotorula* sp. is described as an opportunistic pathogen that causes pathogenesis in immune-suppressed patients with most cases reporting the causal organism to be *R. mucilaginosa.*^3^ However, plant-associated *Rhodotorula* sp. appears to have several beneficial effects on the host rather than any deleterious effects. *Rhodotorula graminis* strain WP1 v1.1 was isolated from poplar as an auxin producing yeast^4^ having significant plant growth promoting (PGP) effects.^5^*Rhodotorula mucilaginosa* (previously *Rhodotorula rubra*) TG-1 was reported to exhibit strong biocontrol activity against fungal and bacterial pathogens upon stem inoculation of rice.^6^ Although several *Rhodotorula* yeasts have started being described from plants, their interaction with plants and their life-strategies have not yet been studied.

*Rhodosporidium* is a sexual, dimorphic red yeast belonging to the family Sporidiobolaceae^7^ that also includes *Rhodotorula*. Haploid yeast cells of *Rhodosporidium* belonging to opposite mating types can conjugate leading to the formation of dikaryotic mycelia.^8^ However, unlike *Rhodosporidium*, *Rhodotorula* is considered to be monomorphic i.e. consisting of the asexual haploid yeast-form with no transition between yeast and filament-forms.^9^ This is the major distinction between the genus *Rhodosporidium* and *Rhodotorula*. The genome sequence of the haploid *Rhodotosporidium toruloides* NP11 has become available.^10^ Several other *Rhodotorula* genomes have been sequenced, such as *R. graminis* strain WP1,^11^ a eurypsychrophilic strain, *R*. sp. JG1b isolated from permafrost in Antarctica,^12^ and *R. mucilaginosa* strain C2.5t1 isolated from cacao seeds.^13^

*Rhodotorula mucilaginosa* strain JGTA-S1 was isolated as an endophyte of *Typha angustifolia*, a wetland macrophyte growing in the metal-contaminated Uranium-mine-associated wetlands in Jaduguda, India.^14^ JGTA-S1 promoted the growth of its natural host, *T. angustifolia*. We undertook a comparative genomics approach to understand the of the yeast *in planta* contributing to its endophytic life-strategy lifestyle and validated at least some of the attributes through experimental approaches that make JGTA-S1 unique. We sequenced, assembled, annotated the genome of JGTA-S1 and compared it to the sequenced genomes of some *Rhodotorula* yeasts and *R. toruloides*. The genome of JGTA-S1 was found to be haploid, with a low repeat content. Comparisons showed all five genomes under study to encode for genes with the potential for benefitting plant growth through phosphate solubilization and phytohormone production. In addition, the genomes appeared to have conserved strategies for adaptation to cold temperatures. The yeasts encoded genes suitable for bypassing plant defence using multiple tactics to facilitate establishment within the plant endosphere. JGTA-S1 was however different from *Rhodosporidium* and *R. graminis* in its nitrogen (N)-metabolism because of a truncated nitrate assimilation pathway which probably led to its association and maintenance of diazotrophic endobacteria leading to complex interaction. JGTA-S1 had an intact mating locus showing that it may undergo a filamentous intermediate in its lifecycle like *Rhodosporidium*.

## 2. Materials and methods

### 2.1. Endophytic colonization of JGTA-S1

*Typha angustifolia* seeds were surface sterilized and grown on artificial soil (Soilrite, Keltech Energies, India) supplemented with 2× Murashige Skoog salt mixture (MS, 15 ml media per 30 g soil) with or without *R. mucilaginosa* culture (10^7^ cfu of JGTA-S1 suspended in 10 ml 1× MS). The yeast was added dropwise around the root of the plants and the plants were grown for 2 months. Shoot lengths were measured for control (*n* = 24) and JGTA-S1-treated (*n* = 24) plants. The significance of the increase in shoot length was tested by Mann-Whitney test and plotted using GraphPad Prism 5. The *Typha* shoots of treated plants 3-weeks post-infection (wpi) were hand sectioned and stained with 0.01% Acridine orange. The sections were observed under a fluorescence microscope (Olympus BX40).

### 2.2. Culture conditions and isolation of genomic DNA

Genomic DNA was isolated from a saturated culture of *R. mucilaginosa* JGTA-S1. Yeast cells were collected by centrifugation from 10 ml saturated culture and was resuspended in 0.5 ml of distilled water. The tube was spun for additional 5 min and the supernatant was discarded. The tube was vortexed briefly to resuspend the pellet in the residual liquid. Two hundred microlitre of a solution containing 2% Triton X100, 1% sodium dodecyl sulphate (SDS), 100 mM NaCl, 100 mM Tris (pH: 8) and 1 mM ethylenediaminetetraacetic acid (EDTA) was added along with 0.2 ml phenol-chloroform and 0.3 g acid-washed glass beads. The tubes were vortexed vigorously for 10 min and 0.2 ml of Tris–EDTA (TE) buffer was added. The tubes were spun for 15 min at 13,000 rpm. The aqueous layer was collected and ethanol precipitated. The pellet was resuspended and treated with 30 µg RNaseA (Himedia) and re-precipitated with 4 M ammonium acetate and 2.5 volumes 100% ethanol at −80°C for 1 h. The tubes were spun at 13,000 rpm for 15 min, washed with 70% ethanol and resuspended in 50 µl TE.

### 2.3. Library preparation and whole genome sequencing

A paired-end library was constructed using the NEXTFlex DNA-sequencing kit (BIOO Scientific, Austin, TX, USA) and sequencing was carried out on Illumina Miseq. The paired-end library had a read length of ∼250 bp with an insert size of 130–580 bp generating 10,499,886 paired-end reads at 262× coverage. Library for MinION was constructed with the 2D library preparation kit, SQK-NSK007, and sequencing was carried out on MinION Mk1b using R9 flowcell (Oxford Nanopore Technologies, Oxford, UK) in a 48 h sequencing protocol on MinKNOW 1.1.20. Reads generated from the run were basecalled using Metrichor V.2.45.1 and converted to fasta format using Poretools.^15^ The long-read library consisted of 74,892 reads containing 850,015,301 bp of 1D and 2D data at 42× coverage.

### 2.4. Genome assembly

Adapters and low-quality bases were trimmed from the raw Illumina reads with Trimmomatic.^16^ Long reads from MinION were error-corrected with Canu.^17^ Whole genome assembly was carried out with SPAdes^18^ using the trimmed and error-corrected reads. Polishing of the final assembly was done with Pilon.^19^ In order to identify the mitochondrial genome, the complete mitochondrial genome of *Rhodotorula taiwanenis* RS1 (Accession no. HF558455.1) was aligned against A2 assembly with NUCmer. To determine the effect of the MinION reads on the assembly, a MinION-free assembly was carried out with SPAdes using only the trimmed Illumina reads. The quality of all assemblies was evaluated with QUAST^20^ and MUMmer.^21^

### 2.5. Ploidy calculation

KmerCountExact programme from BBmap package (https://sourceforge.net/projects/bbmap/) was used to determine the ploidy and size of the genome.

### 2.6. Gene prediction and annotation

Gene prediction was carried out using AUGUSTUS^22^ with protein sequences of strain *Rhodotorula* sp. JG1b as the training dataset. Annotation was carried out in two ways. First, proteins were annotated using Blast2GO.^23^ Second, proteins were queried against NCBI’s nr database using BLASTP searches (E-value < 1e-5) and the annotation of the top hit was fetched from the UniProt database. For Blast2GO, InterProScan was also carried out.^24^ The KEGG-KAAS server (http://www.genome.jp/tools/kaas/) was used for pathway annotation. For prediction of carbohydrate-active-enzymes, protein sequences were submitted to the dbCAN server (http://csbl.bmb.uga.edu/dbCAN/). Annotation of the mitochondrial genome was carried out with MITOS WebServer (http://mitos.bioinf.uni-leipzig.de/index.py). Cluster analysis was carried out with the heatmap.2 function of the gplots package^25^ in R.

### 2.7. Orthologue prediction

For comparison among the *Rhodotorula* and *Rhodosporidium* genomes, protein sequences were extracted from *R*. *mucilaginosa* JGTA-S1, *R. mucilaginosa* C2.5t1, *R*. sp. JG1b, *R. graminis* WP1, and *R. toruloides* NP11. For comparison between the yeasts, endophytes, and pathogens from phylum Basidiomycota, protein sequences were extracted from *Grammothele lineata*, *Serendipita indica, R. graminis* WP1, *Ustilago maydis*, *Puccinia graminis, Melampsora larici-populina, Microbotryum intermedium,* and *Microbotryum lychnidis-dioicae*. OrthoMCL^26^ was run on these sets and analysed using BLASTP (E-value < 1e-5) and an inflation value of 1.5. Enrichment analysis was carried out with clusterProfiler package^27^ in R.

### 2.8. Synteny analysis

Syntenic blocks between *R. mucilaginosa* JGTA-S1 and *R. mucilaginosa* C2.5t1 were identified by MCScanX^28^ and DAGchainer^29^ using a cut-off of five orthologous genes to define a syntenic block.

### 2.9. Phylogeny prediction

A phylogeny based on 3,660 single-copy orthologous proteins was inferred from the OrthoMCL-generated orthologous groups. Each orthologous group of proteins was aligned with ClustalW2^30^ and concatenated into a superalignment. Proml^31^ was used with default parameters to infer a maximum likelihood tree. Similarity among the genomes was inferred from a binary matrix comprising of presence or absence of each orthologous protein. Jaccard distance was used to convert the binary matrix into a distance matrix which was subjected to hierarchical clustering.

### 2.10. Effector prediction

Signal peptide-containing proteins were predicted using SignalP V3.0^32^ (Prediction = ‘Signal peptide’, Y_max_ ≥ 0.5, D score ≥ 0.5, *P* ≥ 0.9). Presence of transmembrane domains and mitochondrial signal peptides in these proteins was predicted using TMHMM V2.0c (http://www.cbs.dtu.dk/services/TMHMM/) and only those proteins with no transmembrane domains were retained in the analysis. TargetP (http://www.cbs.dtu.dk/services/TargetP/) was used to predict subcellular localization. Only those proteins predicted to be secreted were retained (Location = ‘Secreted’). Effectors were predicted from secretory proteins with EffectorP (http://effectorp.csiro.au/).

### 2.11. Nucleotide sequence accession numbers

*Rhodotorula mucilaginosa* JGTA-S1 genome sequencing project has been deposited in NCBI under BioProject PRJNA393004 and BioSample SAMN07313544. The genome assembly has been deposited at DDBJ/ENA/GenBank under the accession PEFX00000000. The Illumina and MinION reads were deposited to Sequence Read Archive under accession numbers SRR5821556 and SRR5821557.

### 2.12. Assay of phosphate solubilization

*Rhodotorula mucilaginosa* JGTA-S1 culture grown for 48 h was spotted on Pikovskaya’s agar plates for qualitative phosphate solubilization assay and halo formation was scored.

### 2.13. Effect of *R. mucilaginosa* on rice plants in presence of low phosphate

Rice seeds (*Oryza sativa* cv. Swarna MTU7029) were surface-sterilized and germinated on filter papers. Four- to five-day-old seedlings of equal lengths were transferred to Soilrite^TM^ containing artificial soil supplied with low-phosphate media [1 µm NaH_2_PO_4_ (as only phosphate source), 2.0 mM NH_4_NO_3_, 1.9 mM KNO_3_, 0.3 mM CaCl_2_.2H_2_0, 0.15 mM MgSO_4_.7H_2_0, 5 µm KI, 25 µm H_3_BO_3_, 0.1 mM MnSO_4_.H_2_O, 0.3 mM ZnSO_4_.7H_2_0, 1 µM Na_2_MoO_4_.2H_2_0, 0.1 µM CuSO_4_.5H_2_0, 0.1 µM CoCl_2_.6H_2_O, 0.1 mM FeSO_4_.7H_2_0, 0.1 mM Na_2_EDTA.2H_2_0, inositol (10 mg l^−^^1^), and Glycine (0.2 mg l^−^^1^)] as described before.^33^ For treated plants, 10^7^ cfu of JGTA-S1 cells suspended in low-phosphate media was added to the soil around a plant root. In control plants, an equal volume of low-phosphate media was supplied. Pictures were taken 10 days post-infection.

### 2.14. Assay of cold tolerance of *R. mucilaginosa* JGTA-S1

*Rhodotorula mucilaginosa* JGTA-S1 and *Saccharomyces cerevisiae* ThyAP4 cells were grown in YPD media to saturation. The cells were spotted on YPD agar at OD_600_=1.0. The cells were incubated at 30 and 5°C and growth was observed after 4 days.

### 2.15. Real-time PCR of anti-freeze genes

JGTA-S1 cells were spotted on tryptic soy broth (TSB) media and grown at 30°C for 4 days. Control cells were frozen in liquid nitrogen after 4 days. For cold treatment, the second batch of cells was shifted from 30 to 5°C and incubated at 5°C for 4 additional days. Primers for anti-freezing genes g5846.t1 (Forward 5’ GGTCTTAAGCCGGTCGTCAA3’ and Reverse 5’AAGGTGGGAGGGGGATAGTC3’) and g5876.t1 (Forward 5’TCGTGTTCCACAGGCTACTG3’ and Reverse 5’TACGGGTCGTTTTCGCAGTT3’) were designed by Primer-BLAST software^34^ using *Rhodotorula* sp. JG1b as an organism. For RNA isolation, cell pellets were suspended in urea buffer (urea 8 M, NaCl 0.5 M, Tris 20 mM, EDTA 20 mM, SDS 2%, pH 8). Glass beads and an equal volume of Trizol (Invitrogen) was added to the suspended cells and subjected to vigorous vortexing. Cells were kept in ice every 2 min. Chloroform was added (one-fifth volume) and vortexed again. The solution was kept on ice for 10 min and centrifuged. An equal amount of Trizol was again added to the supernatant and kept in ice for another 5 min. The chloroform step was repeated. The aqueous layer was transferred and precipitated with 50% (v/v) isopropanol and glycogen (1 µg/ml). The RNA pellet was washed with 85% ethanol and dissolved in RNase-free water. RNAs were subjected to DNase treatment and converted to cDNA with M-MuLV reverse transcriptase and random hexamers (Thermo Scientific) as per the manufacturer’s protocol. An equal amount of cDNA was used for Real-time PCR. A 10 µl of qRT-PCR reaction contained 5 µl DyNAmoColorFlash SYBR Green I master mix (Thermo Scientific), 0.5µM of each primer and 50 ng (Nanodrop 2000, Thermo scientific) of cDNA. Real-time PCR was performed using Step one plus real-time PCR machine (Applied Biosystems). The thermal step-up was 10 min at 95°C, followed by 40 cycles of 15 s at 95°C, 1 min at 60°C. Data analysis was performed using ExpressionSuite Software v1.0.4 (Applied Biosystems). The fold change was calculated by 2^−ΔΔCt^ method using JGTA-S1 glyceraldehyde 3-phosphate dehydrogenase (GAPDH) gene (Fwd 5’ GAGTTGATGCGCGAGCTGGAGAACG 3’ and Rev 5’ GTGGCCGAGAAGGGTCGGCCGAGG 3’) as housekeeping gene. The primer efficiencies were checked and found to be >90 for every primer pairs using LinReg PCR 2017.1.0.0 software. The result corresponds to two independent experiments each in triplicate.

### 2.16. Effect of removal of endosymbiotic bacteria from JGTA-S1 and real-time PCR of 16SrDNA specific DNA

JGTA-S1 was streaked five times in presence of Timentin (250 µg/ml) and Tetracycline (10 µg/ml) on N-free media (5 g Glucose; 0.1 g yeast extract; 0.15 g CaCl_2_; 0.005 g Na_2_MoO_4_; 0.2 g MgSO_4_.7H_2_0; 0.04 g FeSO_4_ 7H_2_0; 0.5 g K_2_HPO_4_; 1.0 g CaCO_3_; 15 g Agar; distilled water to 1 l; pH 7.0) with 5 g/l (NH_4_)_2_SO_4_. Every time, isolated single colonies were used for streaking. Almost 30 colonies were cultured in TSB and genomic DNA was isolated as described earlier. The genomic DNA was used as a template to screen for colonies where no amplification was observed for *Pseudomonas stutzeri* 16SrDNA (Fwd 5’AGGCGACGATCCGTAACTGG3’ and Rev 5’GTCATCCCCACCTTCCTCCG3’). The PCR cycle was 5 min at 98°C followed by 35 cycles of 30 s at 98°C, 15 s at 62°C, 27 s at 72°C with a final extension of 7 min at 72°C. A strain (Strain 11 henceforth) lacking *P. stutzeri* 16SrDNA amplification in two independent PCR reactions was used for further studies. Strain 11 or JGTA-S1 control cells were grown in TSB or N-free media containing (NH_4_)_2_SO_4._ The cell pellets were washed twice with water and suspended in water. The OD_600_ was normalized and spotted on TSB and N-free media at different dilutions [undiluted (0.5 OD), 1:100 and 1:1,000]. For qPCR genomic DNA was isolated from JGTA-S1 (control) and Strain 11. *Pseudomonas stutzeri* 16SrDNA gene specific primers (Fwd 5’ TGAGTAATGCCTAGGAATCTGCC 3’ and Rev 5’ ATCCGACCTAGGCTCATCTGATA 3’) were designed using Primer-BLAST software using *Pseudomonas stutzeri* DSM 4166 as an organism for qPCR. An equal amount of genomic DNA was used for Real-time PCR. A 10 µl of qRT-PCR reaction contained 5 µl DyNAmoColorFlash SYBR Green I master mix (Thermo Scientific), 0.5 µM of each primer and 1.5 ng (measured in Qubit, Thermo Fisher) of genomic DNA. Real-time PCR was performed using a 7500 real-time PCR machine (Applied Biosystems). The thermal step-up was 10 min at 95°C, followed by 40 cycles of 15 s at 95°C, 30 s at 60°C. The fold change was calculated by 2^−ΔΔCt^ method using JGTA-S1 GAPDH gene as housekeeping gene. The result corresponds to three independent experiments each performed in triplicate. The significance of the increase in relative *P. stutzeri* levels/JGTA-S1 cell was tested by Mann-Whitney test and plotted using GraphPad Prism 5. The primer efficiencies were checked using LinReg PCR 2017.1.0.0 software as described before. A Strain 11.2 was generated from Strain 11. Strain 11 was subjected to one passage in presence of Carbenicillin (100 µg/ml), Gentamycin (30 µg/ml), and Kanamycin (50 µg/ml) in N-free media + (NH_4_)_2_SO_4_. The colonies were revived once by growing them on TSB plate w/o antibiotics followed by another round of streaking in N-free media + (NH_4_)_2_SO_4_ containing Carbenicillin, Gentamycin, and Kanamycin. A primer pair corresponding to the V3 region of 16SrDNA of bacteria (Ba341Fwd: CCTACGGGAGGCAGCAG and Ba518Rev: ATTACCGCGGCTGCTGG) was used for amplification of total endobacteria. Genomic DNA (1 µg, measured by Nanodrop, Thermo scientific) from control, Strains 11 and 11.2 was used as a template. For no template control (NTC) no DNA was added. The PCR cycle was 5 min at 94°C, followed by 11 cycles of 1 min at 94°C, 1 min at 65°C, 3 min at 72°C and 20 cycles of 1 min at 94°C, 1 min at 55°C, 3 min at 72°C and a final extension at 72°C for 10 min. Spotting assay (on N-free media) and qPCR from Strain 11.2 were done as described for Strain 11.

## 3. Results and discussion

### 3.1. *Typha* endophyte *R. mucilaginosa* JGTA-S1 has a compact repeat-poor haploid genome

**Figure 1:**
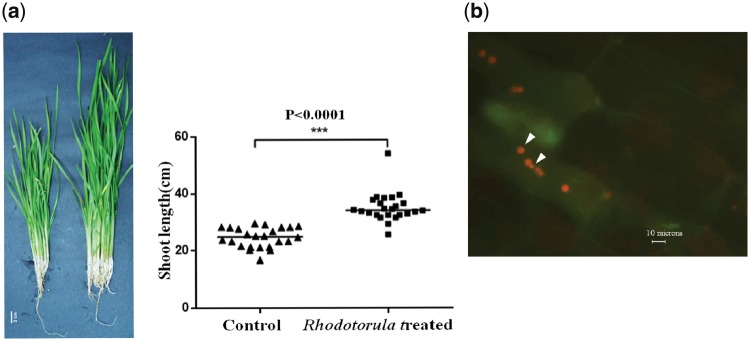
*Rhodotorula mucilaginosa* JGTA-S1 colonizes in *T. angustifolia* endophytically and promotes growth. *Typha angustifolia* seeds were surface sterilized and germinated on artificial soil. JGTA-S1 culture was added around the root. For control plants, 1× MS was added. Plant length was measured after 2 months. Plant lengths were subjected to the Mann-Whitney test. For determination of colonization, *Typha* shoots were hand sectioned and stained with Acridine orange 3wpi. (a) Digital image of control and JGTA-S1-treated *Typha* (left), Mann-Whitney test for shoot length (right). (b) *Rhodotorula* cells in shoot sections of *Typha*. Color figures are available at *DNARES* online.

**Figure 2:**
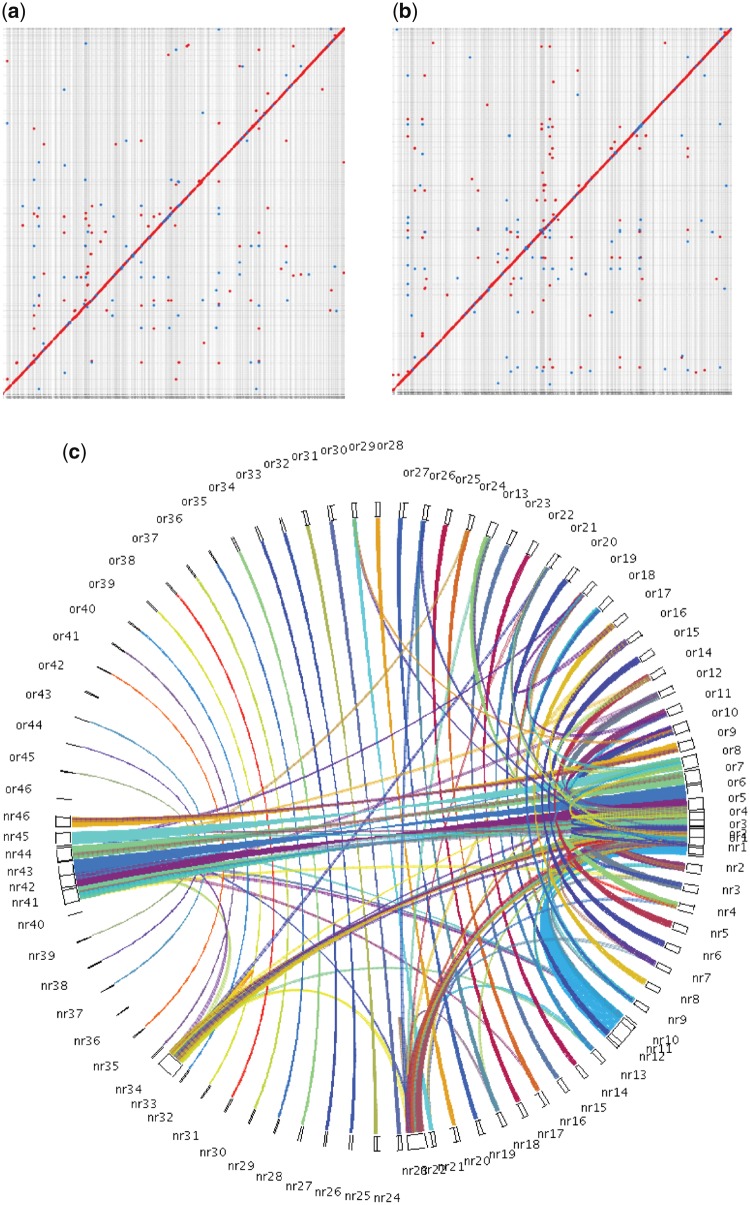
Genome synteny between *R. mucilaginosa* JGTA-S1 and *R. mucilaginosa* C2.5t1. (a) Dot plot showing nucleotide vs nucleotide alignment of JGTA-S1 genome assembly (Y-axis) compared with C2.5t1 genome assembly (X-axis) was generated by the NUCmer programme from the MUMmer 3.23 package.^20^ (b) Dot plot showing alignment after six-frame translations of JGTA-S1 genome assembly (Y-axis) and C2.5t1 genome assembly (X-axis) was generated by the PROmer programme from the MUMmer 3.23 package.^20^ Red dots indicate matches from the same strand and blue dots indicate matches from opposite strands. The straight red line indicates good alignment between the X and Y axis. (c) Collinear blocks between JGTA-S1 (labelled as ‘or’ in the top half of semi-circle) and C2.5t1 (labelled as ‘nr’ in the bottom half of semi-circle) were identified by MCScanX^28^ from all-against-all BLASTP searches between JGTA-S1 and C2.5t1. The circle plotter script in MCScanX^28^ was used to generate the plot. Each rectangle depicts a scaffold in the genome and the synteny between scaffolds is depicted as ribbons. Color figures are available at *DNARES* online.

*Rhodotorula mucilaginosa* JGTA-S1 was isolated as an endophyte of *T. angustifolia*. JGTA-S1 was isolated from *Typha* shoot. An independent isolate of *R. mucilaginosa* (JGTA-R1, KU051691.1) was isolated from *Typha* root demonstrating that the yeast was probably able to colonize both shoot and root of *Typha*. The yeast showed strong growth promoting effects on *Typha* (Fig. 1a). The increase in shoot lengths of JGTA-S1-treated plants was tested with Mann-Whitney analysis and the median value was found to be significantly increased upon yeast treatment (Fig. 1a, right panel). The endophytic colonization of JGTA-S1 was confirmed by staining shoot sections of *Typha* infected with JGTA-S1 with acridine orange 3 wpi. JGTA cells were found in *Typha* sections validating its endophytic colonization (Fig. 1b). To get a better insight into its life-strategy, JGTA-S1 was sequenced on Illumina Miseq at 262× coverage and Oxford Nanopore MinION at 42× coverage. Self-corrected MionION reads were combined with adapter-trimmed and quality-checked Illumina reads to generate a hybrid assembly. Use of Nanopore reads in addition to the Illumina reads resulted in a better assembly comprising of fewer but longer scaffolds and almost 3-fold increased N50 value (Supplementary Table S1) resulting in Assembly A1 (Table 1). This was further improved by removing scaffolds that were <2,000 bp to generate assembly A2 that consisted of only 46 scaffolds, N50 of 685,833, length of 20,108,097 bp where the largest scaffold was 2,159,349 bp (Table 1). KmerCountExact was also able to determine that JGTA-S1 was a haploid genome (Supplementary Fig. S1) of size 21,822,513 bp. JGTA-S1 assembly size was close to the estimated size. Moreover, it had only 46 scaffolds in comparison to the 1, 034 scaffolds of *R*. *mucilaginosa* strain C2.5t1. MUMmerplots of NUCmer and PROmer alignments showed colinearity between JGTA-S1 and C2.5t1 suggesting a good agreement between the genomes (Fig. 2a and b). The mitochondrial genome of JGTA-S1 was integrated into Scaffold 2 of the assembly (Supplementary Fig. S2). The annotated mitochondrial genome consisted of tRNA, rRNA, genes encoding ATP synthase subunits and cytochrome oxidase subunits (Supplementary Table S2).

**Table 1:** Assembly statistics of *R. mucilaginosa* JGTA-S1

Features	A1 assembly	A2 assembly
No of scaffolds	341	46
N50	685,833	685,833
Length of assembly	20,358,951 bp	20,108,097 bp
Largest scaffold	2,159,349 bp	2,159,349 bp

In the absence of annotations from C2.5t1 the only sequenced *mucilaginosa* genome, we used the genome and proteome of *R.* sp. JG1b for training AUGUSTUS.^22^ A total of 5,922 protein-coding genes were predicted from the genome of *R. mucilaginosa* JGTA-S1 (Table 2). The average gene lengths, mean exon lengths and mean intron lengths were determined to be 2,778, 275, and 134 bp, respectively (Table 2). It was found to average 200 genes Mb^−1^ with the densest region having ∼350 genes Mb^−1^ which is within the range reported for Puccinomycotina genomes.^35^ The same gene prediction protocol resulted in 6,463 predicted genes in *R. mucilaginosa* C2.5t1 (Table 2). The UniProt and Blast2GO annotations of JGTA-S1 are presented in Supplementary Table S3. Among the top 5 GO terms in ‘Biological Process’ were those involved in the N-metabolic process (Supplementary Fig. S3).

**Table 2:** Gene prediction statistics for *R. mucilaginosa* JGTA-S1 and C2.5t1

Features	*R. mucilaginosa* JGTA-S1	*R. mucilaginosa* C2.5t1
No. of protein-coding genes	5,922	6,463
No. of predicted CDSs	42,105	43,928
No. of predicted introns	36,215	37,981
Average gene length	2,778 bp	2,453 bp
Average CDS length	275 bp	263 bp
Average intron length	134 bp	112 bp
Max gene length	25,521 bp	18,172 bp
Min gene length	208 bp	95 bp
Max intron length	7,824 bp	3,480 bp
Min intron length	42 bp	6 bp
Average number of intron/ protein-coding gene	6.12	5.88
Max CDS length	4,641 bp	4,509 bp
Min CDS length	3 bp	3 bp

CDS: Coding sequence.

The previously reported C2.5t1 genome had numerous scaffolds, so the C2.5t1 genome was scaffolded using an in-house assembler with the JGTA-S1 genome as a reference assembly (https://github.com/madhubioinfo/STLab-assembler). This assembler aligns scaffolds to a reference genome using length cut-off of 1,500 bp (–maxgap = 500 –mincluster = 100) and >97% identity to eliminate the poorly aligned region. The scaffolds were then ordered based on the reference assembly and the gaps were filled with Ns. Subsequently, the scaffolded assembly of C2.5t1 also rendered to 46 scaffolds making it easier to compare synteny (Fig. 2c and Supplementary Table S4). Forty-four pairs of scaffolds containing 5,359 gene pairs were syntenic between JGTA-S1 and C2.5t1. The two pairs of scaffolds that did not contain significant synteny, i.e. Scaffold 42 (Supplementary Fig. S4a, Scaffold 42 on Y axis and corresponding scaffold of C2.5t1 on X axis) and Scaffold 46 of JGTA-S1 (Supplementary Fig. S4b, Scaffold 46 on Y axis and corresponding scaffold of C2.5t1 on X axis) were small scaffolds (40,000 and 8,000 bp in length) and were most likely caused by a small inversion and a deletion. Overall a one-to-one relationship was observed between pairs of scaffolds of the two genomes again confirming that JGTA-S1 and C2.5t1 were closely related and represented haploid genomes.

**Figure 3:**
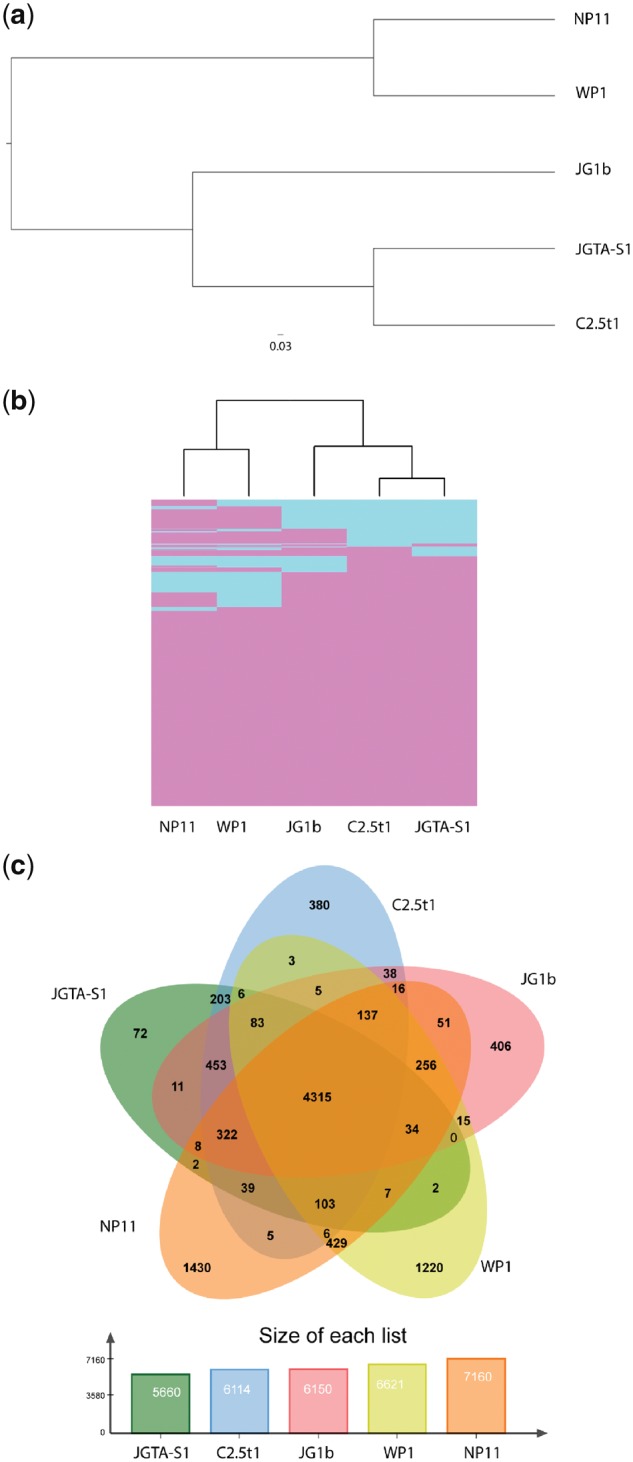
Relatedness of *R. mucilaginosa* with other closely related red yeasts. *Rhodotorula mucilaginosa* JGTA-S1 (JGTA-S1) genome was compared with C2.5t1 (*R. mucilaginosa* C2.5t1), WP1 (*R. graminis* WP1), JG1b (*R.* sp JG1b) and NP11 (*R. toruloides* NP11). (a) Maximum likelihood tree based on the concatenated alignment of 3,660 single-copy orthologous groups. OrthoMCL^26^ was used to infer orthologues among the *Rhodotorula* genomes. Proteins of each orthologous group were aligned with ClustalW2^30^ and concatenated into a superalignment and used for phylogeny prediction. (b) Hierarchical clustering based on the presence or absence of orthologous gene families. A binary matrix consisting of the presence or absence of orthologous gene families was converted into a distance matrix and subjected to hierarchical clustering. The heatmap shows presence (pink) and absence (blue) of orthologues. (c) Shared and unique orthologue gene families among the five genomes are shown in the Venn diagram. Color figures are available at *DNARES* online.

The genome of JGTA-S1 was compared with the whole genome sequences of four other *Rhodotorula* genomes and *R. toruloides* (Table 3). *Rhodotorula graminis* WP1 was found to endophytically colonize poplar.^5^*Rhodotorula mucilaginosa* strain C2.5t1 was isolated from cacao seeds.^13^*R*. sp JG1b was isolated from permafrost in Antarctica.^36^*Rhodosporidium toruloides* (previously *Rhodotorula glutinis*) was also found in association with the woody structure of plants.^37^ Thus *Rhodosporidium* and *Rhodotorula* yeasts appeared to have a tendency for plant-association although except for *R. graminis* strain WP1 none of the other strains have been experimentally demonstrated as endophytes neither have their life strategies been demonstrated in plants. The completeness of the assembly of JGTA-S1 was assessed with BUSCO and compared with the other genomes (Table 3). Thus, the assembly of JGTA-S1 was comparable to the assemblies of the other genomes. Overall the *Rhodotorula* genomes and the *Rhodosporidium* were ∼20 MB in size. The genome size of this group was small when compared with other classes in order Pucciniomycotina (Supplementary Table S5). This reduction in genome size appears to be a feature common to class Microbotryomycetes. The relationship among the five *Rhodotorula* genomes was assessed with phylogeny and cluster analysis. A total of 34,483 proteins from the five organisms were clustered into 6,774 groups by OrthoMCL. Phylogeny was inferred from 3,660 single-copy orthologues and showed that JGTA-S1 and C2.5t1 were most closely related to the eurypsychrophilic organism *R*. sp. JG1b (Fig. 3a). This was compared with a cluster analysis on the presence–absence of all orthologues (Fig. 3b). The two trees had the same topography indicating that the gene content of these organisms reflected their evolutionary history that was largely unaffected by factors such as horizontal gene transfer. Core orthologues defined as orthologues that were present in all five organisms consisted of 4,315 proteins (Fig. 3c) enriched in 88 GO IDs. These core proteins belonged to various biosynthetic pathways, DNA replication machinery, signalling pathways (Supplementary Fig. S5a) and were located in the mitochondria (Supplementary Fig. S5b) and functioned as helicases, transferases, and GTPases (Supplementary Fig. S5c). Thus, the core proteome mostly had housekeeping functions. Also included in the core proteome were several plant hormones synthesis pathways, such as cytokinin, auxin, and lipid biosynthesis genes such as isocitrate dehydrogenase and malic enzyme (Supplementary Table S6) (discussed below). Thus, all genomes appeared to have host-beneficial and lipid-producing capabilities. The *R. mucilaginosas*, JGTA-S1 and C2.5t1, and JG1b had smaller genomes and encoded mostly core proteins having very few unique orthologues, i.e. 72 in JGTA-S1, 380 and 406 in C2.5t1 and JG1b respectively. Most of these 72 JGTA-S1 proteins had unknown functions or shared no similarity with any known proteins. Among the proteins having annotations, gag-pol polyproteins that are part of retro elements were a major class (Supplementary Table S7).

**Table 3:** Genomes used in the comparative study and their genome statistics

Genomes	Source	Size of genome (MB)	N50 value of the assembly	Complete BUSCOs	Fragmented BUSCOs	Missing BUSCOs	Number of protein- coding genes
*R. mucilaginosa* JGTA- S1	Stem of *T. angustifolia*, India	20.11	685,833	269	10	11	5,922
*R. mucilaginosa* C2.5t1	Seeds of *Theobroma cacao*, Cameroon	19.98	45,031	255	25	10	6,463
*R. sp* JGIb	Permafrost, Antarctica	19.39	301,937	274	4	12	6,681
*R. graminis* WP1	Stem of *Populus trichocarpa U.S.A*	21.01	1,420,730	271	4	15	7,278
*R. toruloides* NP11	Unknown	20.22	574,942	275	4	11	8,140

Only 2% of these genomes were occupied by repeat elements (Supplementary Table S8). A higher number of long terminal repeat (LTR) elements (Copia, Gypsy) were found in the genomes of JGTA-S1 (57), C2.5t1 (97), and JG1b (73) in comparison to NP11 (34) and WP1 (26) while a large number of LINES were found in NP11. Overall the LTR repeat content of these genomes was low like that of the corn smut pathogen, *U. maydis.*^38^

### 3.2. Comparison with other endophytes and pathogens

**Figure 4:**
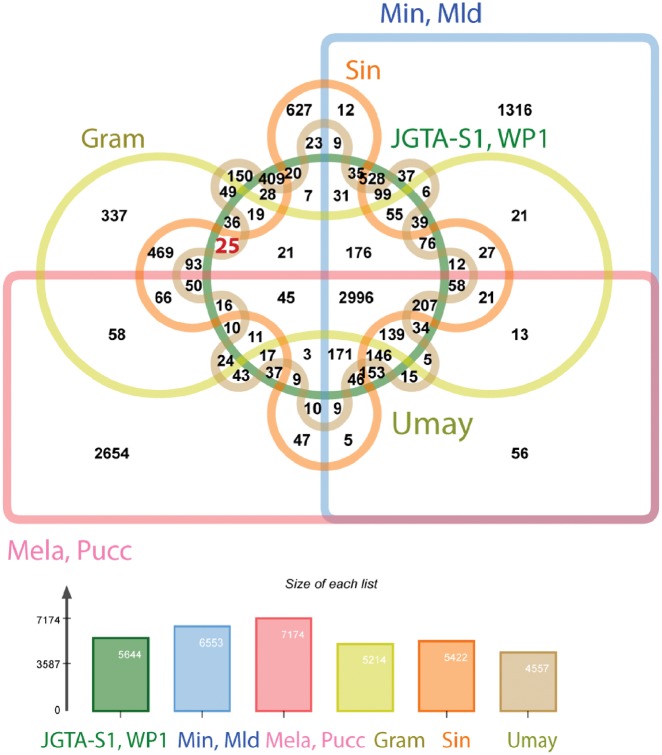
Comparison of orthologues of *R. mucilaginosa* JGTA-S1 with other Basidiomycetous fungi. OrthoMCL^26^ was used to infer orthologues for *R. mucilaginosa* JGTA-S1, Umay (*U. maydis*), Min (*M. intermedium*), Mld (*M. lychnidis-dioicae*), Gram (*G. lineata*), JGTA-S1 (*R. mucilaginosa* JGTA-S1), WP1 (*R. graminis* WP1), Sin (*S. indica*), Mela (*M. larici-populina),* and Pucc (*P. graminis*). Shared and unique orthologue families are shown in the Venn diagram. To make visualization simple, closely-related genomes have been grouped together. Color figures are available at *DNARES* online.

To determine what genes were common between JGTA-S1 and other endophytes an orthologue analysis was carried out with known endophytes from Basidiomycota. *Grammothele lineata*—an endophyte that colonizes jute,^39^*S. indica* (previously *Piriformospora indica*)—a root-colonizing endophyte^40^ and *R. graminis* WP1^41^—a poplar endophyte were used in the study. In contrast, pathogens such as the corn smut biotroph, *U. maydis*,^42^ wheat stem rust biotroph, *P. graminis*,^43^ the poplar rust biotroph*, M. larici-populina*,^44^ and two closely related biotrophs, *M. intermedium,* and *M. lychnidis-dioicae*^45^ were included. The Venn diagram shows that 2,996 orthologue groups were shared by all 9 species but only 25 groups were shared exclusively by *Rhodotorula* (JGTA-S1 and WP1) and the endophytes (*G. lineata* and *S. indica*) (red highlight in Fig. 4). These 25 endophyte-specific groups included proteins such as NmrA-domain-containing proteins (Supplementary Table S9). JGTA-S1 encoded 6 such NmrA proteins. NmrA is a transcriptional regulator involved in regulating N-metabolism when different N-sources are available.^46^ In addition, *Rhodotorula* and other members of Pucciniomycotina lacked a gene cluster homologous to 19 A coding for secreted effectors in *U. maydis*^47^ and some other Ustilaginomycotina. The deletion of the 19 A cluster genes in *U. maydis* led the mutant to behave like an endophyte. However, in absence of sufficient homology in the region flanking the 19 A cluster of *U. maydis* with *Rhodotorula* sp. it is difficult to correlate the lack of 19 A cluster with the endophytic lifestyle of *Rhodotorula*. On the other hand, *Rhodotorula* shared a high number of orthologues, i.e. 528 with the pathogens *M. intermedium* and *M. lychnidis-dioicae* both of which belong to the same class as *Rhodotorula*, i.e. Microbotryomycetes. A predominant class of genes common to the Microbotryomycetes was the proteophosphoglycans. These are glycoproteins that in the intercellular human pathogen, *Leishmania donovani* have been shown to help in attaching to host cell during infection.^48^ This group of proteins appeared to have undergone expansion in *Rhodotorula*. JGTA-S1 had 85 copies of proteophosphoglycan ppg4 and 18 copies of proteophosphoglycan ppg5. A search for ‘proteophosphoglycan AND basidiomycetes’ ‘[porgn:__txid5204]’ in the NR database resulted in 1887 hits (search done on 10.05.18). Of those, 81% were hits to *Rhodotorula*. The role of proteophosphoglycan in fungi is unclear but they may be involved in host cell invasion.

### 3.3. *Rhodotorula* yeasts have genes for cold adaptation

**Figure 5:**
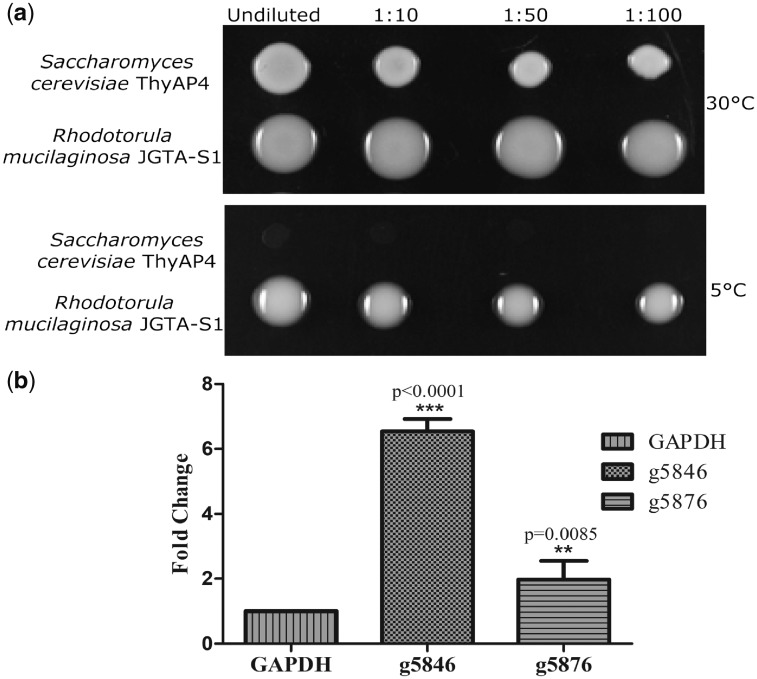
Cold tolerance of *R. mucilaginosa* JGTA-S1. JGTA-S1 and *S. cerevisiae* ThyAP4 cells were grown in YPD medium till saturation and adjusted to an O.D. of 1. The cells were serial diluted and spotted on YPD plates and incubated at 30 and 5°C for 4days. For RNA isolation JGTA-S1 cells were grown at 30°C for 4 days followed by 4 days at 5°C. Control cells were frozen after 4 days of growth at 30°C. Real-time PCR was done with two putative anti-freezing genes. Results are from two independent experiments done in triplicate. (a) JGTA-S1 and *S. cerevisiae* ThyAP4 cells spotted on YPD (b) Expression of g5846 and g5876 compared with JGTA-S1 GAPDH as housekeeping gene.

Many genera within the class Microbotryomycetes can tolerate cold. These include *Rhodotorula*, *Rhodosporidium*, *Leucosporidium*, *Microbotryum,* and *Sporobolomyces.*^2^ Since JGTA-S1 genome showed strong relatedness to the eurypsychrophilic *Rhodotorula* sp. JG1b that was isolated from the McMurdo Dry Valleys of Antarctica,^34^ our genomes were searched for the presence of cold tolerance genes. All five *Rhodotorula* genomes were found to contain multiple copies of genes encoding putative anti-freeze glycoproteins. These are g574.t1, g736.t1, g5846.t1, g5847.t1, g5875.t1, and g5876.t1 in JGTA-S1 and their orthologues in other genomes are listed in Supplementary Table S10. These proteins found in cold-adapted organisms are known to prevent ice crystal formation enabling growth at low temperatures.^49^ The ability of *R. mucilaginosa* JGTA-S1 to withstand low temperature was tested by growing it at on YPD plates at 5°C. JGTA-S1 showed much better growth at 5°C in comparison to *S. cerevisiae* strain ThyAP4 4-days post-spotting (Fig. 5a). In accordance with this data, two randomly chosen anti-freezing genes g5846.t1 and g5876.t1 were found to be upregulated 6.6- and 1.9-fold, respectively in a Real-time PCR experiment when JGTA-S1 was grown at 5°C compared with control cells grown at 30°C (Fig. 5b). This result showed that JGTA-S1 was cold-tolerant and the two anti-freezing genes studied were functional. Since the cold-tolerant genes were found both in JGTA-S1 as well as in JG1b, the genes could have similar functions in the other red yeasts as well.

### 3.4. *Rhodotorula* has a reduced set of CAZY enzymes

**Figure 6:**
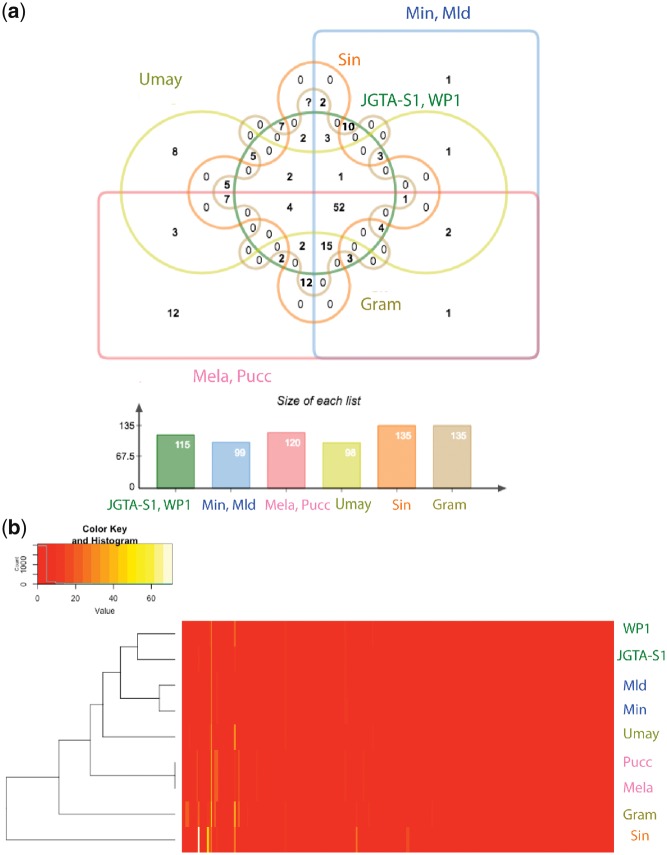
CAZY enzymes of *R. mucilaginosa* JGTA-S1 in comparison with other Basidiomycetous fungi. CAZY enzymes were inferred from the proteome of JGTA-S1**,** Umay (*U. maydis*), Min (*M. intermedium*), Mld (*M. lychnidis-dioicae*), Gram (*G. lineata*), WP1 (*R. graminis* WP1), Sin (*S. indica*), Mela, (*M. larici-populina*), and Pucc (*P. graminis*). (a) Shared and unique CAZY enzymes are shown in the Venn diagram. To make visualization simple, closely related genomes have been grouped together. (b) Comparison of CAZY profile of JGTA-S1 to other Basidiomycetous fungi by cluster analysis based on of number of CAZY genes per genome. Heatmap shows gene counts per CAZY family. Color figures are available at *DNARES* online.

Overall, JGTA-S1 had only 271 CAZY enzymes (Supplementary Table S11). In order to look for CAZY enzymes that are specific for endophytic function, we compared CAZY families in JGTA-S1 with those of *G. lineata*, *S. indica* and *R. graminis* WP1, *U. maydis*, *P. graminis, M. larici-populina, M. intermedium,* and *M. lychnidis-dioicae*. Interestingly, clustering based on copy numbers of CAZY genes showed that the *Rhodotorulas* (JGTA-S1 and WP1) were more similar in CAZY profile to *M. intermedium* and *M. lychnidis-dioicae* than to endophytes *S. indica* and *G. lineata* (Fig. 6b). Only two CAZY families were unique to the endophytes, carbohydrate-binding module 1 (CBM1) and glycosyl hydrolase 88 (GH88) (Fig. 6b). These profiles were present only in JGTA-S1, WP1, *S. indica,* and *G. lineata*. CBM1 is a protein domain that binds to polysaccharides and is found in fungal hydrolytic enzymes that degrade plant cell wall. Larroque *et al*.^50^ found many CBM1-domain-containing proteins in necrotrophic and hemibiotrophic fungi but no CBM1-domains was found in biotrophic fungi. Our study showed that endophytes also have CBM1 domains and may indeed be more similar to necrotrophs than to biotrophs. It is relevant to mention here that *S. indica* despite being a PGP fungus was reported to cause host cell death during mutualistic symbiosis.^51^ Some CAZY families were present in higher numbers in JGTA-S1 such as GT90, GH16 (Supplementary Table S11). The GT90 proteins were associated with capsule formation in encapsulated yeasts.^52^ GH16 enzymes catalyse the breakdown of β-1, 3-1, 4-glucans^53^ commonly found in the cell walls of grasses. JGTA-S1 encodes GH16 enzymes that possibly facilitate penetration and establishment in grasses such as *Typha* and rice. Several cellulases such as cello biohydrolases (GH6 and GH7), endoglucanases (GH45, GH74) were missing in JGTA-S1. Only glucosidase (GH3) could be detected. Also missing from JGTA-S1 were hemicellulases such as xylanases (GH10 and GH11), arabino uranosidases (GH51, GH54, and GH62), endoarabinase (GH43 and GH93), and xyloglucanase (GH74 and GH12).

### 3.5. *Rhodotorula* yeasts encode multiple strategies for establishment and evasion of plant defence

Chitin deacetylases of family CE4 were present in JGTA-S1 and the other *Rhodotorula* and *Rhodosporidium* genomes (Supplementary Table S11). This enzyme converts fungal chitin to chitosan that helps in avoiding detection by the host.^54^ Thus, CE4 might play a role in the evasion of plant defence in these organisms.

Another way of avoiding chitin-based plant immune response is to scavenge degraded chitin molecules. Fungal LysM domain-containing proteins are chitin-binding proteins that scavenge the degraded chitin molecules that form during fungal penetration preventing activation of plant defence.^55^ CBM1 proteins described in the section above are LysM domain-containing enzymes. Ten CBM1 proteins were predicted in JGTA-S1 possibly supporting the ability of the fungus to evade plant defence.

All the *Rhodotorula* genomes studied here were found to have at least one copy of salicylate hydroxylase, a key enzyme required for breaking down of salicylic acid (SA), a plant defence hormone that is produced both locally as well as systemically upon encounter with microbe-associated molecular patterns^56^ triggering plant defence against biotrophs. Salicylate hydroxylase has been reported in the smut fungus *U. maydis* where it may play a role in biotrophic growth.^57^ Copies of salicylate hydroxylase were encoded on all genomes such as KWU46987.1 (*R.* sp JG1b), XP_018270700.1 (*R. graminis* WP1), g3206.t1, g5580.t1 (C2.5t1), EMS24466.1 (NP11) and g1003.t1, g1030.t1 (JGTA-S1). JGTA-S1 also contained several genes of the Jasmonic acid pathway (g718.t1, g2844.t1, and g4607.t1:Supplementary Table S12). Jasmonic acid is an alternate plant defence hormone known to have an antagonistic effect on SA-mediated plant defence.^58^

### 3.6. *R. mucilaginosa* encodes genes related to PGP

JGTA-S1 genome contained many genes that could potentially influence plant hormonal response. Genes coding for the auxin pathway were found in JGTA-S1. Three genes of the abscisic acid pathway were also found on JGTA-S1. The gene, g288.t1-encoded isopentenyl-diphosphate delta-isomerase that is required to convert plant hormone cytokinin from inactive to an active form^59^ and tRNA-dimethylallyl transferase the first gene in the cytokinin biosynthetic pathway (g5608.t1). The yeast also had the potential to influence Brassinosteroid pathway as it contained several genes required for sterol biosynthesis. JGTA-S1 also coded genes for (R)-acetoin and (R, R)-2, 3-butandiol production that are volatiles produced by many PGP rhizobacteria. All genes related to hormone production are listed in Supplementary Table S12.

### 3.7. Solubilization and uptake of phosphate

**Figure 7:**
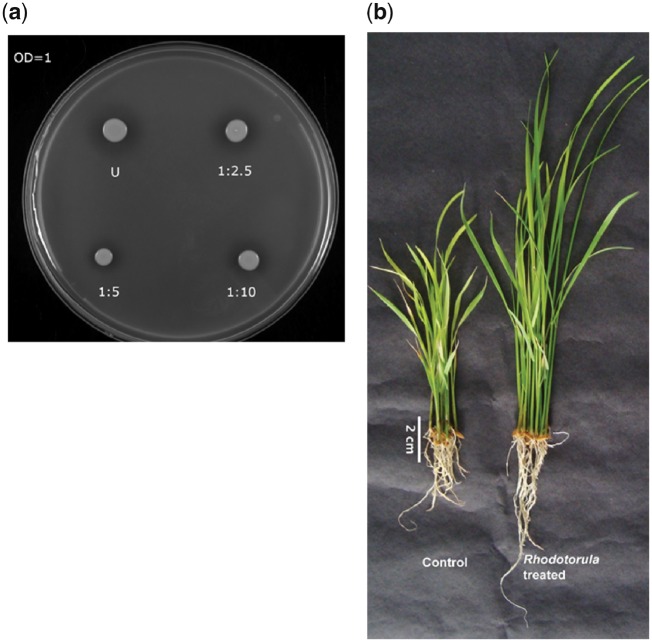
Phosphate solubilization by *R. mucilaginosa* JGTA-S1. JGTA-S1 cells were grown in TSB broth overnight. The O.D. was adjusted to 1 and cells were diluted and spotted on Pikovskaya’s media. For studying the effect of JGTA-S1 on plant phosphate nutrition, rice plants grown on low-phosphate media were supplemented with JGTA-S1. (a) The halo around the JGTA-S1 spots indicates solubilization of phosphate on Pikovskaya’s media. (b) The growth of rice in low-phosphate media in control and *Rhodotorula*-treated condition 10dpi. Color figures are available at *DNARES* online.

The release of phosphate from inorganic or organic sources and its uptake is an important way by which endophytes benefit plants.^60^ JGTA-S1 contained organic phosphatases (g2241.t1, g4636.t1), inorganic phosphatases (g1099.t1, g3530.t1, g5792.t1, and g2815.t1) and phosphate transporters (g981.t1, g4895.t1, g2031.t1, g660.t1, and g5404.t1) (Supplementary Table S3). We experimentally validated the phosphate solubilization ability of JGTA-S1. Clear zones around JGTA-S1 growth were observed on Pikovskaya’s agar plates (Fig. 7a) which indicated its ability to solubilize insoluble calcium phosphate present in the media. Consistent with this data, JGTA-S1 was able to improve growth in rice plants grown in low-phosphate media (Fig. 7b) in comparison to untreated controls which turned chlorotic 10 days after transfer in low-phosphate media.

### 3.8. Effector proteins of JGTA-S1

EffectorP was used to predict effectors from secretory proteins. Of the 1, 134 candidate effector proteins identified from the five genomes, only 278 proteins (24.5%) could be annotated by BLASTP against nr database (E value < 1e-05) (Supplementary Table S13) which was not surprising since most effector proteins do not contain domains.^61^ The effectors were clustered into 223 groups containing 499 proteins while 635 proteins remained as singletons, which mean that most effectors were unique to each organism. Moreover, no conserved motifs could be identified in the effectors. This observation reiterates the finding that fungal effectors are often species-specific and are evolving at a rapid pace such that orthologues of effectors are not seen in closely related genomes.^62^ The annotated proteins included chitin deacetylase that functions in modifying chitin as mentioned previously, a malic enzyme involved in the fatty acid synthesis and a serine carboxypeptidase. Also found among the effectors were some proteophosphoglycan proteins (Supplementary Table S13).

### 3.9. *Rhodotorula* encodes an intact mating locus

**Figure 8:**
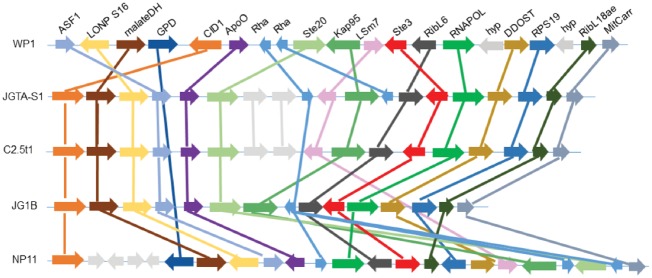
Synteny in the MAT locus of the *Rhodotorula* genomes. The MAT locus of WP1was annotated. The gene names are indicated and its synteny is compared with other genomes. Each arrow represents a gene. Homologous genes have the same colour and are connected by lines. Color figures are available at *DNARES* online.

The mating locus of fungi is responsible for sex determination and filament formation. It consisted of homeodomain transcription factors and pheromone-related genes. An intact MAT locus on Scaffold 13 of JGTA-S1 was identified with homologues of a map kinase (*STE20*), a pheromone receptor gene (*STE3*), and pheromone precursor genes (*RHA1*, *RHA2*). Another MAT locus containing the homeodomain transcription factors *HD1* and a remnant of *HD2* were identified on Scaffold 7 of JGTA-S1. It was difficult to conclude if Scaffolds 7 and 13 were part of the same chromosome or were unlinked. These loci were at least 500 kb apart. The large distance between the pheromone-related locus and the HD locus in JGTA-S1 was similar to *Mixia osmundae*^35^ and *Sporidiobolus salmonicolor*.^63^ This mating system, therefore, could be bipolar, tetrapolar or pseudobipolar. No polymorphisms of *STE3* and *STE20* genes were found between the *R. mucilaginosa* genomes, suggesting either, (i) JGTA-S1 and C2.5t1 belonged to the same mating type and the opposite mating type is yet to be reported, (ii) JGTA-S1 and C2.5t1 were homothallic and were able to mate with single individual, or that (iii) JGTA-S1 and C2.5t1 were clonal members of an asexually reproducing population. Comparison of synteny in the MAT locus showed good agreement among JGTA-S1, C2.5t, and JG1b (Fig. 8). Sexual life cycle and transition from yeast to filament form are tightly linked phenomena in many dimorphic fungi. Filament formation occurs after mating of complementary cells of opposite mating types leading to the formation of dikaryotic mycelium in *R. toruloides*.^8^ However, no filamentous growth or sexual lifecycle was ever reported in *Rhodotorula* yeasts leading to the segregation of the *Rhodotorula* genus from the sexually reproducing *Rhodosporidium*. Although could not be demonstrated, a possible filamentous stage was also predicted for *R. graminis* WP1.^41^ Presence of a complete set of MAT genes in JGTA-S1 may predict the presence of a filamentous stage similarly, either by the conjugation of yet unidentified complementary mating types or alternatively, from germinating asexual spores as described in Zygomycetes.

### 3.10. JGTA-S1 contain a truncated nitrogen metabolic pathway

Nitrogen is taken up by fungi in its organic form as amino acids and in its inorganic form as nitrate and ammonium. Nitrate is assimilated into ammonium by the nitrate assimilation pathway that consists of a nitrate reductase, nitrite reductase, and nitrate transporters belonging to Major Facilitator Superfamily (MFS). Several rust fungi that are obligate biotrophs such as *Melampsora* and *Puccinia* have lost this pathway.^64^ We found an incomplete nitrate assimilation pathway in JGTA-S1, C2.5t1, and JG1b that was missing nitrite reductase and the MFS nitrate transporters. On the other hand, NP11 and WP1 had an intact nitrate assimilation pathway. This could partly be circumvented in JGTA-S1 by the uptake of ammonium by the two ammonium transporter proteins (g2114.t1 and g5165.t1). One of these, g5165.t1 was an orthologue of a high-affinity ammonium transporter from *U. maydis* that can induce filament formation in low ammonium conditions.^65^ JGTA-S1 also encoded glutamate synthase (g5248.t1) and glutamine synthetase (g5571.t1) the key enzymes involved in ammonia assimilation. In addition, nine amino acid transporters were encoded by JGTA-S1. *Rhodotorula* yeasts have the ability to utilize urea as a sole N-source with a potential to improve urea utilization of plants. This property was confirmed by the genome of JGTA-S1 that encoded urea degrading enzymes and urea transporters (g4941.t1 and g5832.t1).

### 3.11. *R. mucilaginosa* JGTA-S1 may contain endosymbiotic bacteria

**Figure 9:**
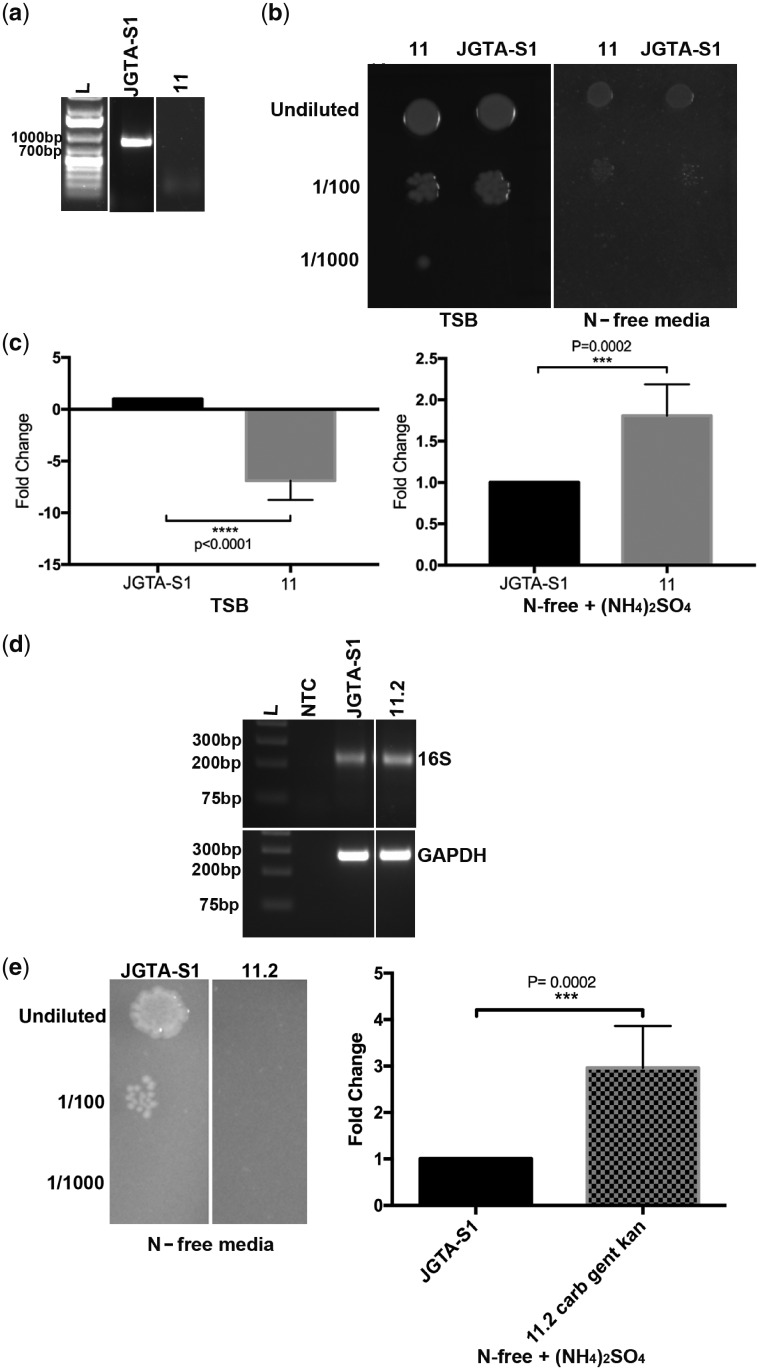
JGTA-S1 cells had reduced growth when the endobacteria were removed. JGTA-S1 cells were streaked in presence of Timentin and Tetracycline to kill the endosymbiotic *P. stutzeri* within JGTA-S1 cells. Single colonies were grown in TSB after five rounds of streaking in presence of antibiotics, genomic DNA was isolated and PCR performed using *P. stutzeri* specific 16SrDNA primers to isolate strains deficient in *P.stutzeri*. Strain 11 grown in TSB or N-free media + (NH_4_)_2_SO_4_ was spotted on TSB or N-free media in different dilutions and compared with control JGTA-S1 cells or used for isolation of genomic DNA for qPCR. Strain 11.2 was generated by further streaking Strain 11 in the presence of Carbenicillin, Gentamycin and Kanamycin and used for genomic DNA isolation and PCR using primers for the V3 region of total bacterial 16SrDNA and qPCR with *P. stutzeri* specific 16SrDNA (a) *P. stutzeri* specific 16SrDNA amplification from Timentin and Tetracycline treated Strain 11 vs control JGTA-S1 confirming reduced level of *P. stutzeri*. (b) Spotting assay of control JGTA-S1 and Strain 11 on TSB and N-free media plate. Undiluted (OD_600_= 0.5) (c) qPCR of Strain 11 and control JGTA-S1 genomic DNA with primers designed to amplify *P. stutzeri* 16SrDNA relative to yeast GAPDH gene. Results are from three independent experiments each performed in triplicate. The significance is calculated by Mann-Whitney’s test. (d) PCR amplification of the V3 region of bacterial 16SrDNA from JGTA-S1 control and 11.2 cells (upper panel). The lower panel shows amplification of JGTA-S1 GAPDH gene. NTC: No template control (e) Spotting assay of control JGTA-S1 and Strain 11.2 on N-free media plate (Left panel). qPCR of Strain 11.2 genomic DNA with primers designed to amplify *P. stutzeri* 16SrDNA relative to yeast GAPDH gene (right panel).

Another way in which JGTA-S1 could circumvent the absence of the complete nitrate assimilation pathway is by association with N-fixing bacteria*. Rhodotorula mucilaginosa* JGTA-S1 was able to grow in N-free media (Fig. 9b). Growth in N-deprived media had earlier been reported in *U. maydis* where an association with an N-fixing bacterium living as an endosymbiont was assumed to supply N to the host.^66^ At least four dinitrogen reductase sequences corresponding to diazotrophs were amplified from JGTA-S1 genomic DNA (MG566093–MG566096). To search for bacteria in the genomic data the Illumina reads were mapped to the final assembly of JGTA-S1 and only the unmapped reads (1.85%) were retained to search for bacterial sequences. Several bacterial sequences could be found within the 15,047 pairs of reads. Out of these, 50 reads having over 98% identity to *Pseudomonas stutzeri* were found (Supplementary Table S14). The results corroborated with the finding that two *nifH* sequences MG566093 and MG566095 corresponding to *P. stutzeri*—an N-fixing as well as the denitrifying bacterium was amplified from JGTA-S1 genomic DNA. A *P. stutzeri* strain was experimentally confirmed to be able to colonize endosymbiotically in *R. mucilaginosa* (Saha *et al.,* unpublished). Although several reads belonging to other N-fixing bacteria *Klebsiella* sp., *Bradyrhizobium* sp., *Mesorhizobium loti*, *Rhizobium* sp. and *Sinorhizobium* sp., etc. were also found (Supplementary Table S14), no single bacterium dominated the reads prompting us to speculate (i) low diazotroph load in the genomic DNA of JGTA-S1 or (ii) the presence of several different bacteria. This observation is also supported by the low number of endosymbiotic bacteria/fungal cell (or spore) reported in *S. indica* and mycorrhizal fungus *Gigaspora margarita*, N-fixing bacteria comprising even a smaller fraction of this small number.^67^ For a recently sequenced genome of an obligatory Mollicutes-related endosymbiont (DhMRE) from Arbuscular mycorrhiza *Dentiscutata heterogama* the spores of the fungus were enriched before the endobacterial genome was isolated and sequenced.^68^ A protocol that enriched the DhMRE sequence and removed mycorrhizal DNA was developed for this purpose. We did not opt for any such enrichment procedures which might explain why the bacterial sequence counts of *P. stutzeri* (or other diazotrophs) were low. Since the presences of the other diazotrophs were unconfirmed, *P. stutzeri* was given prime importance for further studies. To investigate the importance of the endosymbionts we streaked JGTA-S1 several times in presence of Tetracycline and Timentin chosen based on the sensitivity of a *P. stutzeri* strain (JGTA-R3) isolated in the laboratory.^14^ After five passages, JGTA-S1 colonies were screened for the absence of *P. stutzeri* specific 16SrDNA amplification. A strain (Strain 11) showing no amplification of *P. stutzeri* 16SrDNA from the JGTA-S1 genomic DNA in two independent PCR reactions was used for further experiments (Fig. 9a). Spotting assay was performed on TSB (complete media) and N-free media as a measure of growth or fitness of JGTA-S1 cells. In parallel, qPCR was performed where *P. stutzeri* 16SrDNA level was quantitated and normalized against JGTA-S1 GAPDH gene. This relative abundance reflected the number of *P. stutzeri* per JGTA-S1 cell. Strain 11 did not show any significant reduction in growth compared with control JGTA-S1 in spotting assay (Fig. 9b). The qPCR result indicated that the number of *P. stutzeri*/JGTA-S1 was lower in Strain 11 compared with control when the Strain 11 was grown on TSB media as expected (Fig. 9a and c, left panel). The results were however completely different when ammonium was used as the sole N-source. *Pseudomonas stutzeri* levels/JGTA-S1 cell in Strain 11 was 1.8-fold higher than control when the cells were grown in N-free media with (NH_4_)_2_SO_4_ (Fig. 9c, right panel). This result showed that the levels of *P. stutzeri* alone did not dictate the fitness of JGTA-S1, especially on N-free media. This was not surprising since JGTA-S1 appeared to contain diazotrophs other than *P. stutzeri* underlining the possibility that the other diazotrophic endobacteria compensated for the loss of *P. stutzeri* (Supplementary Table S14 and Fig. 9d). The relative levels of *P. stutzeri/*JGTA-S1 in Strain 11 showed an increase compared with JGTA-S1 control cells when ammonium was the sole N-source compared with when it was grown in complete media. This indicated that (i) yeast growth in spotting assay and the number of *P. stutzeri*/JGTA-S1 cells did not always correlate and there was a more complex interplay (ii) The number of *P. stutzeri*/JGTA-S1 in Strain 11 depended on whether N was available in organic plus inorganic forms (TSB) or just as inorganic form (ammonium) indicating a possible correlation between *P. stutzeri* levels/yeast cell and N-nutrition of JGTA-S1. Endobacteria were found to be capable of multiplying within mycorrhiza at different stages of the fungal development.^67^ Whether this relative increase in *P. stutzeri*/JGTA-S1 was a consequence of multiplication of *P. stutzeri* within JGTA-S1 in response to inadequate N-availability is an interesting proposition to study in future. However, a JGTA-S1 strain (Strain 11.2) derived from Strain 11 grown in TSB media contained endobacteria despite treatment with five different antibiotics Timentin, Tetracycline, Carbenicillin, Gentamycin, and Kanamycin. A primer pair targeted to amplify the V3 region of bacterial 16SrDNA gene was used with JGTA-S1 and Strain 11.2 genomic DNA as a template for showing the total endobacteria (Fig. 9d, upper panel). Strain 11.2 was grown in presence of ammonium as sole N-source in presence Carbenicillin, Gentamycin, and Kanamycin as it failed to grow in presence of all five antibiotics. Strain 11.2 was then spotted on N-free media or the genomic DNA was used for qPCR to measure *P. stutzeri* levels/JGTA-S1 cell as before. Strain 11.2, however, had a significant fitness cost unlike Strain 11 (Fig. 9e, left panel) in spotting assay probably due to the removal of additional diazotrophs. Despite that, the *P. stutzeri* levels/JGTA-S1 cell was increased in Strain 11.2 under the N-inadequate condition, that too, even strongly than Strain 11 further confirming that this increase was a surveillance strategy which was not to be correlated with JGTA-S1 fitness especially under N-inadequate condition. The results can be explained by the fact that endobacteria numbers/fungal cell or spore reported for *S. indica* (2–20/fungal cell) and *Candidatus Glomeribacter gigasporarum* (10/spore) are tightly regulated.^67^ In *Rhodotorula*, despite being a survival strategy increase of *P. stutzeri* levels/JGTA-S1 cell beyond a point may adversely affect the fitness of the fungus.

## 4. Conclusion

Our results, therefore, provide a genomic perspective into the lifecycle of *Rhodotorula mucilaginosa* JGTA-S1 shedding some light into its ability to invade and improve plant growth and nutrition. The effect of JGTA-S1 on the plant phosphate nutrition, hormonal response, and defence was clear from relevant genes present in the JGTA-S1 genome. JGTA-S1 had a truncated N-assimilation pathway but was still able to grow in absence of nitrogen. This was potentially due to its ability to maintain diazotrophs inside the yeast. These endobacteria could not be cured and their levels/yeast cells were under complex regulation depending on the N-regime under which the yeast was grown. Comparative analyses of various endophytic and pathogens fungi within Basidiomycota showed that although the *Rhodotorula*s were evolutionarily closer to the hemibiotrophs *M. intermedium* and *M. lychnidis-dioicae* they shared some genes required for host invasion exclusively with other endophytes.

## Supplementary Material

Supplementary Data
