# Supplementary material for: A unique life-strategy of an endophytic yeast Rhodotorula mucilaginosa JGTA-S1—a comparative genomics viewpoint
Source: DNA Res. 2019 Jan 7;26(2):131–46. doi: 10.1093/dnares/dsy044 (PMC6476726; doi:10.1093/dnares/dsy044)
Supplement: Supplementary Data [file dsy044_supp.zip › dsy044-Suppl_data/dsy044_Supplementary_data.docx]

**Figure S1.** ***R. mucilaginosa* JGTA-S1 is a haploid genome**

Kmer histogram (k=31) of whole genome Illumina reads showing a single peak at 137X coverage indicated haploid nature of *R. mucilaginosa* JGTA-S1 genome.

**Figure S2**. **The mitochondrial genome of JGTA-S1**

The complete mitochondrial genome of *Rhodotorula taiwanenis* RS1 was (X-axis) aligned to scaffold 2 of the JGTA-S1 assembly (Y-axis) with NUCmer in MUMmer 3.23 package showing that mitochondrial genome of JGTA-S1 is included in scaffold 2 of assembly. Red dots indicate matches from the same strand and blue dots indicate matches from opposite strands.

**Figure S3**. **Top 20 GO terms for protein sequences of JGTA-S1**

GO ontology of the JGTA-S1 proteins for BP (biological process), CC (cellular component) and MF (molecular function) categories were found by Blast2GO.

**Figure S4**. Analysis of synteny between *R. mucilaginosa* JGTA-S1 and C2.5t1

MUMmer plots were used to show scaffolds of JGTA-S1 that were not syntenic to C2.5t1. (a) scaffold 42 of JGTA-S1 (Y-axis) aligned with scaffold 36 of C2.5t1 (X-axis). (b) scaffold 46 of JGTA-S1 (Y-axis) aligned with scaffold 40 of C2.5t1 (X-axis). Red dots indicate matches from the same strand and blue dots indicate matches from opposite strands.

**Figure S5.** **GO Enrichment of the core proteome of *Rhodotorula* genomes**

ReviGo plots was used for GO Enrichment analysis of the core proteome of *Rhodotorula* genomes for (a) biological process, (b) cellular component and (c) molecular function. Each circle is a GO term and size of circle indicates the number of genes that have it. The significance is shown by the color key in the top right.
