# Supplementary figures and images for: A unique life-strategy of an endophytic yeast Rhodotorula mucilaginosa JGTA-S1—a comparative genomics viewpoint
Source: DNA Res. 2019 Jan 7;26(2):131–46. doi: 10.1093/dnares/dsy044 (PMC6476726; doi:10.1093/dnares/dsy044)

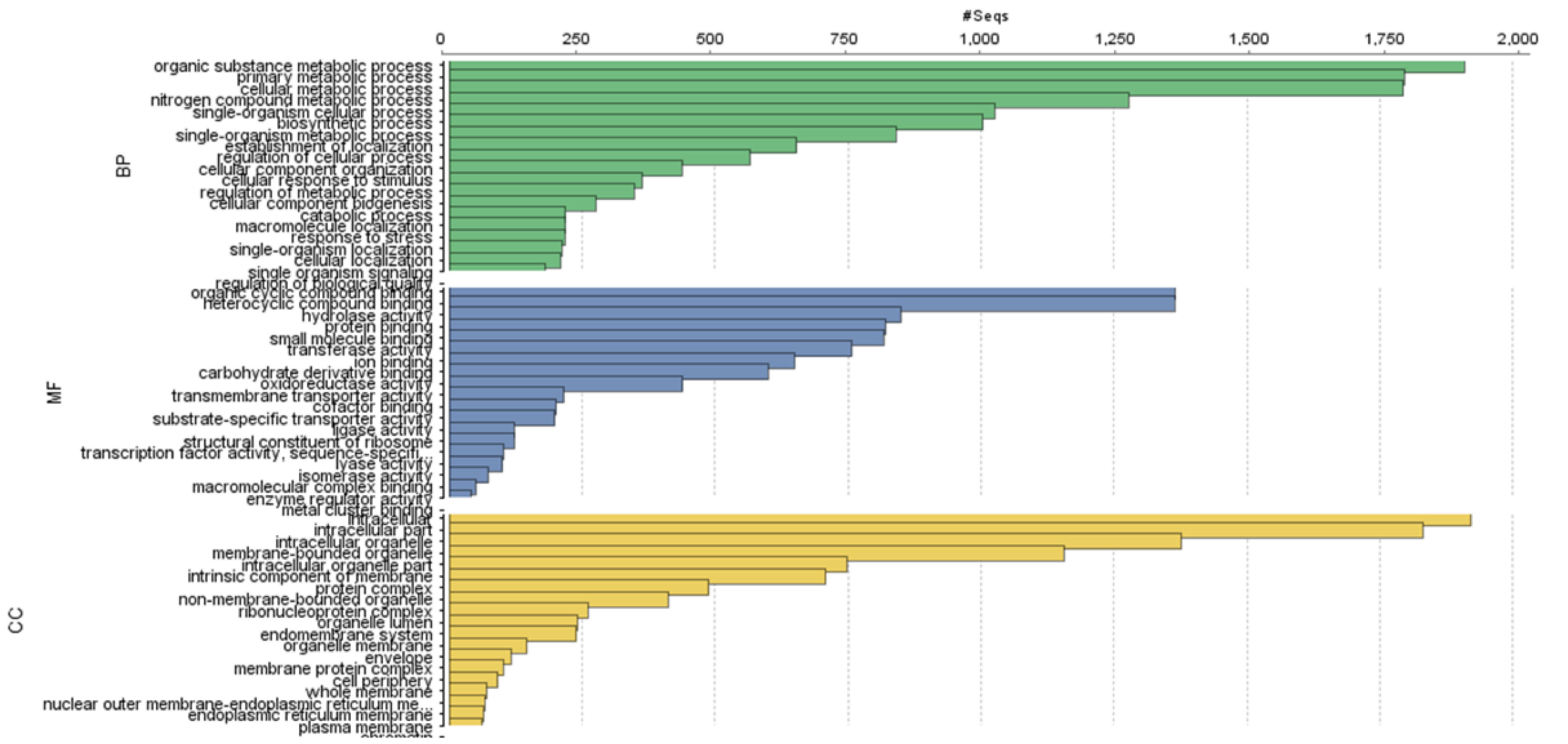

Supplement: Supplementary Data [file dsy044_supp.zip › dsy044-Suppl_data/dsy044_Figure_S3.tif]

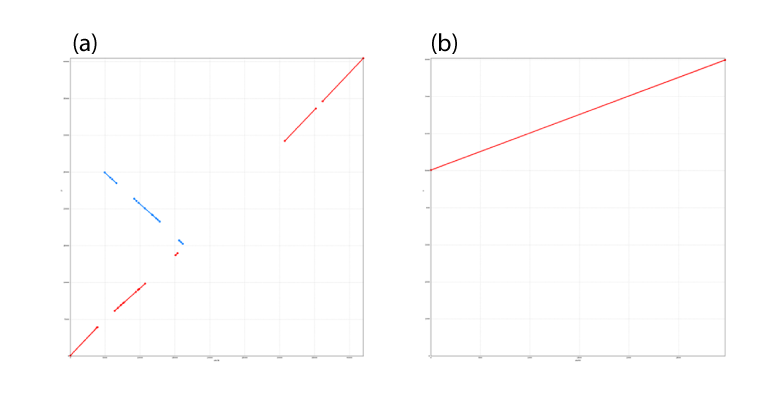

Supplement: Supplementary Data [file dsy044_supp.zip › dsy044-Suppl_data/dsy044_Figure_S4.tif]

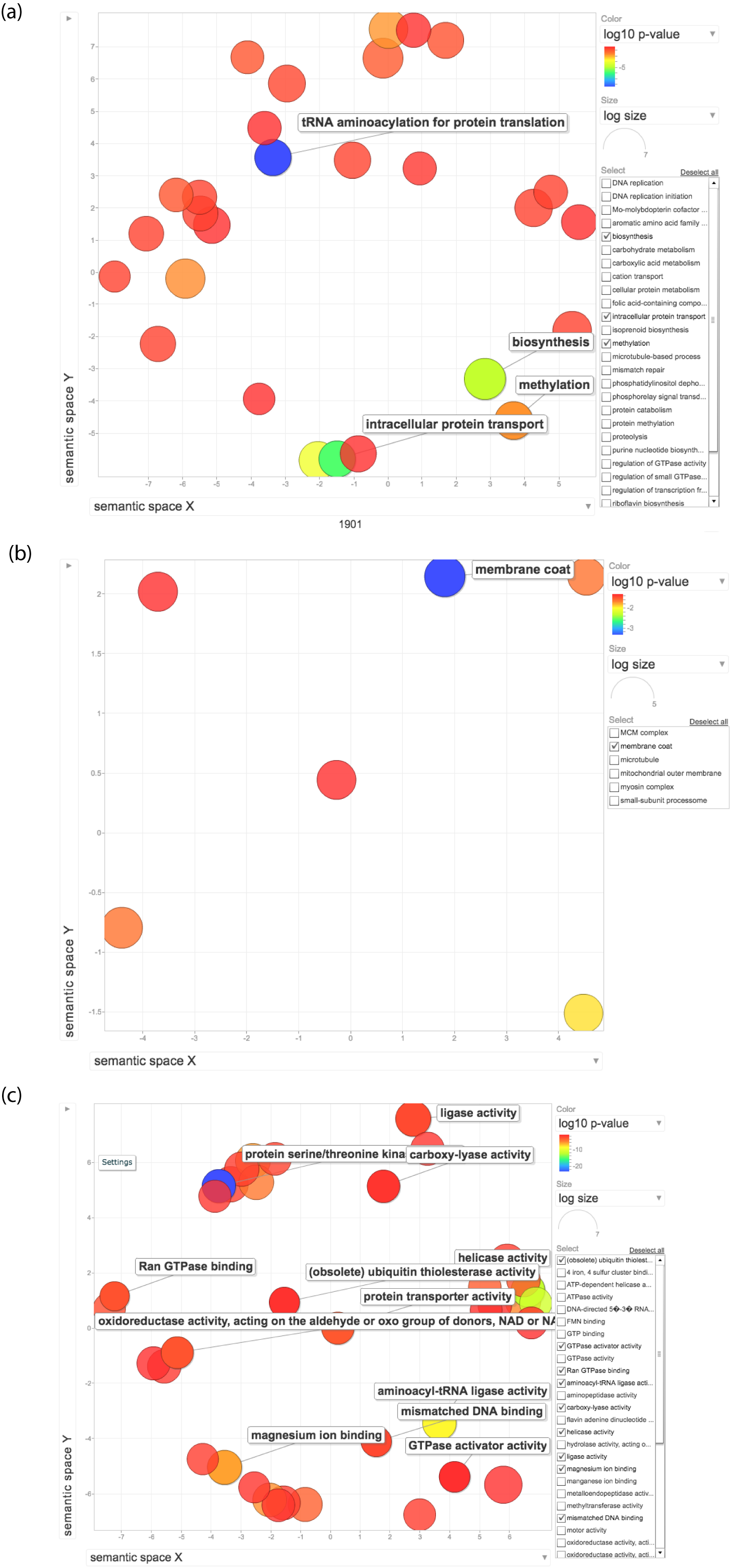

Supplement: Supplementary Data [file dsy044_supp.zip › dsy044-Suppl_data/dsy044_Figure_S5.tif]

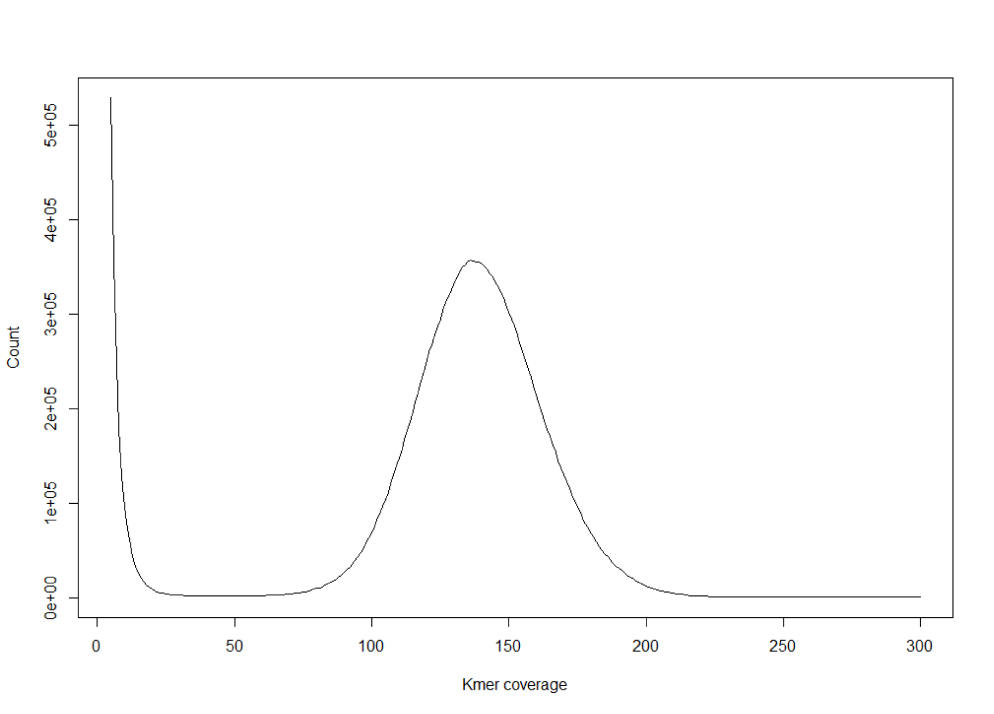

Supplement: Supplementary Data [file dsy044_supp.zip › dsy044-Suppl_data/dsy044_Figure_S1.tif]

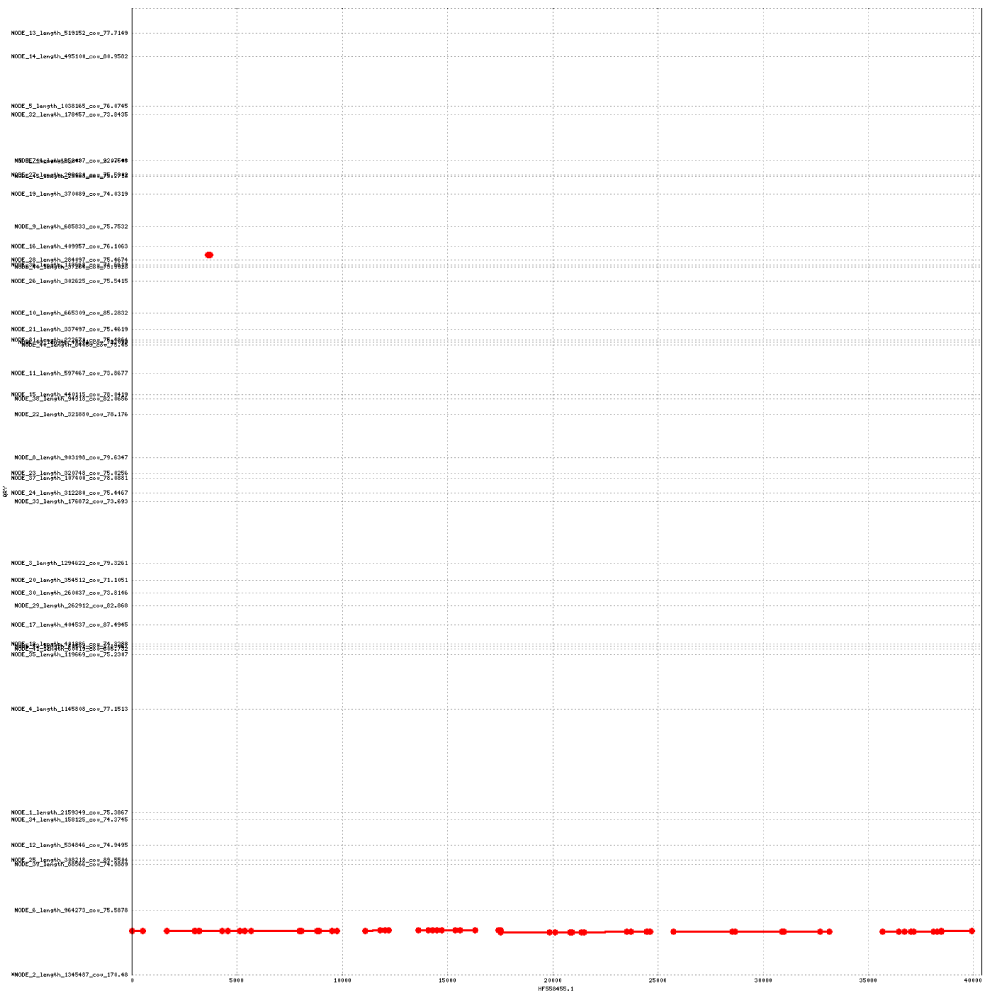

Supplement: Supplementary Data [file dsy044_supp.zip › dsy044-Suppl_data/dsy044_Figure_S2.tif]
